# Identification of cytochrome CYP2E1 as critical mediator of synergistic effects of alcohol and cellular lipid accumulation in hepatocytes *in vitro*

**DOI:** 10.18632/oncotarget.6203

**Published:** 2015-10-20

**Authors:** Abdo Mahli, Wolfgang E. Thasler, Eleonora Patsenker, Sebastian Müller, Felix Stickel, Martina Müller, Helmut K. Seitz, Arthur I. Cederbaum, Claus Hellerbrand

**Affiliations:** ^1^ Department of Internal Medicine I, University Hospital Regensburg, Regensburg, Germany; ^2^ Grosshadern Tissue Bank and Center for Liver Cell Research, Department of Surgery, Ludwig-Maximilians-University Munich, Munich, Germany; ^3^ Department of Clinical Research, University of Bern, Murtenstrasse, Bern, Switzerland; ^4^ Centre for Alcohol Research, University of Heidelberg and Department of Medicine (Gastroenterogy), Salem Medical Centre, Heidelberg, Germany; ^5^ Department of Pharmacology and Systems Therapeutics, Icahn School of Medicine at Mount Sinai, New York, NY, USA

**Keywords:** alcohol, steatosis, autophagy, CYP2E1, Pathology Section

## Abstract

Clinical studies propose a causative link between the consumption of alcohol and the development and progression of liver disease in obese individuals. However, it is incompletely understood how alcohol and obesity interact and whether the combined effects are additive or synergistic. In this study, we developed an *in vitro* model to address this question. Lipid accumulation in primary human hepatocytes was induced by incubation with oleic acid. Subsequently, steatotic and control hepatocytes were incubated with up to 50 mM alcohol. This alcohol concentration on its own revealed only minimal effects but significantly enhanced oleate-induced lipogenesis and cellular triglyceride content compared to control cells. Similarly, lipid peroxidation, oxidative stress and pro-inflammatory gene expression as well as CYP2E1 levels and activity were synergistically induced by alcohol and steatosis. CYP2E1 inhibition blunted these synergistic pathological effects. Notably, alcohol and cellular steatosis also induced autophagy in a synergistic manner, and also this was mediated *via* CYP2E1. Further induction of autophagy ameliorated the joint effects of alcohol and oleic acid on hepatocellular lipid accumulation and inflammatory gene expression while inhibition of autophagy further enhanced the dual pathological effects. Further analyses revealed that the joint synergistic effect of alcohol and steatosis on autophagy was mediated *via* activation of the JNK-pathway. In summary, our data indicate that alcohol induces not only pathological but also protective mechanisms in steatotic hepatocytes *via* CYP2E1. These findings may have important implications on the prognosis and treatment of alcoholic liver disease particularly in obese individuals.

## INTRODUCTION

Chronic alcohol consumption is one of the main etiological factors for liver disease worldwide; however, only a fraction of drinkers develop significant hepatic inflammation known as alcoholic steatohepatitis (ASH), and even less progress to significant hepatic fibrosis and cirrhosis. Still, alcoholic liver disease (ALD) is the second most common reason for liver transplantation in the United States and Europe, which clearly indicates the need to unravel the mechanisms and factors promoting hepatic injury in ALD [1, 2].

More recently, the pathophysiological significance of hepatic lipid accumulation in the absence of significant alcohol consumption is increasingly recognized. Thus, nonalcoholic fatty liver disease (NAFLD) is now consider ed the most common cause of liver enzyme elevations in Western countries [3]. NAFLD is regarded as the hepatic manifestation of the metabolic syndrome, characterized by central obesity and insulin resistance, resulting in diabetes type 2, dyslipidemia, and hypertension [4]. Similarly to ALD, NAFLD encompasses a wide range of pathological conditions from mild hepatic steatosis to steatohepatitis (nonalcoholic steatohepatitis [NASH]) with significant necroinflammation and progressive fibrosis. In its advanced form, NASH is believed to account for a large fraction, if not entirely for what was previously termed “cryptogenic cirrhosis” [5].

Several studies suggest that hepatic steatosis alone can prime the liver to progress to more serious pathologies upon its exposure to subsequent metabolic stressors such as alcohol [6, 7]. Carmiel-Haggai *et al.* showed that short-term binge alcohol exposure increased apoptosis and liver injury in obese rats compared to lean controls [8]. Recently, we have shown that chronic alcohol application in combination with a high fat diet significantly enhanced hepatic inflammation and fibrosis compared to alcohol or high fat diet alone [9]. Also clinical studies suggest a strong causative link between alcohol consumption and progressive liver disease in individuals with high fat-intake or obesity. Obese alcoholics have an accentuated elevation in serum transaminase levels [10, 11]. Furthermore, in subjects with heavy alcohol consumption, obesity is an independent risk factor for the development of both acute alcohol-induced hepatitis and cirrhosis [12, 13]. Even moderate alcohol consumption leads to an increase of liver enzymes with rising body mass index (BMI) [14]. Moreover, both elevated BMI and alcohol consumption are related to the progression of chronic liver disease, with evidence of a synergistic interaction between the two factors [15].

Alcohol as well as dietary lipids are predominantly metabolized in hepatocytes, rendering the interactions between alcohol- and lipid-metabolism very likely. Alcohol undergoes enzymatically-catalyzed oxidative metabolism to acetaldehyde by alcohol dehydrogenase (ADH) and microsomal cytochrome P450 CYP2E1. The resulting acetaldehyde is further oxidized by acetaldehyde dehydrogenase (ALDH) to acetate which leads ultimately to lipid accumulation, reactive oxygen species (ROS) formation and induction of oxidative stress [16]. On the other hand, hepatocellular lipid accumulation impairs the oxidative capacity of mitochondria and stimulates peroxisomal and microsomal pathways of fat oxidation, leading to additional oxidative stress that triggers production of inflammatory cytokines [17, 18]. However, it is incompletely understood how alcohol and obesity interact and whether the combined effects on the progression of liver injury are additive or synergistic.

The present study aimed to establish an *in vitro* model to study the effect of moderate alcohol levels on steatotic hepatocytes and to unravel the interacting effects between hepatocellular lipid accumulation and alcohol metabolism.

## RESULTS

### Effects of alcohol and oleic acid on hepatocellular lipid synthesis and accumulation

**Figure 1:**
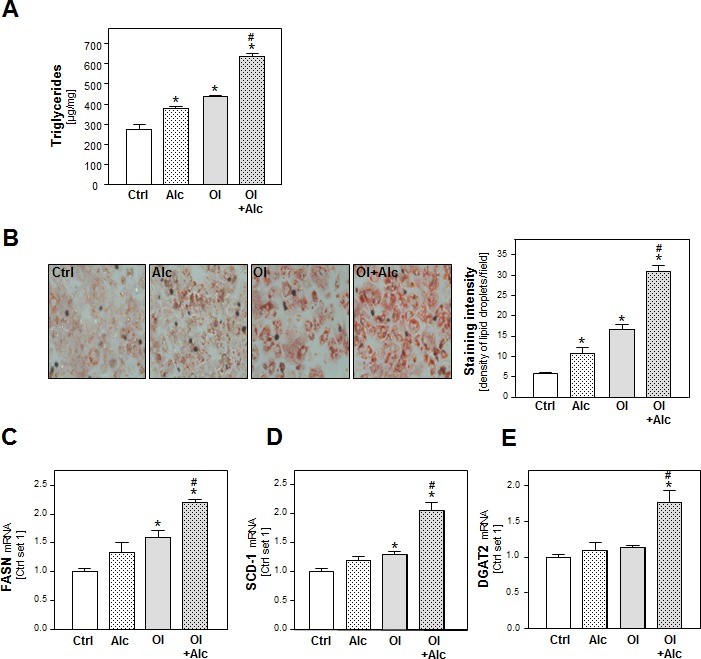
Analysis of the effect of alcohol and oleate on hepatocellular lipid metabolism and lipid accumulation PHH were pre-incubated with 0.2 mM oleate (Ol) or BSA (served as control [Ctrl]) for 24h. Subsequently, cells were co-incubated with 50 mM alcohol (Alc) for additional 24h. **A.** Cellular triglyceride content normalized to total cellular protein. **B.** Representative images of cells stained with Oil red O (left panel); quantification of staining intensity of lipid droplets using a light microscope with digitalized camera and a Metaview image analysis system. The density of lipid droplets per microscopic area was calculated from four different fields (right panel). Analysis of mRNA levels of **C.** FASN, **D.** SCD-1 and **E.** DGAT2 by quantitative RT-PCR (*: *p* < 0.05 compared to control; #: *p* < 0.05 compared to oleate or alcohol condition).

To study the combined effect of alcohol and oleic acid (oleate) on PHH, we chose an oleate concentration of 0.2 mM and an alcohol (Alc) concentration of 50 mM. We have previous shown that hepatocellular steatosis induced by this oleate concentration led to pathological alterations similar to those found in hepatic tissue of NAFLD-patients [19, 20]. Furthermore, previous *in vitro* studies have revealed that *in vitro* exposure of hepatic cells to 50 mM alcohol mimics the situation of “moderate” alcohol consumption in humans [21-23]. In these concentrations, neither oleate or alcohol alone nor the alcohol and oleate combination affected the viability of PHH *in vitro* within 48h (Suppl.Figure S1A-C). Analysis of cellular triglyceride (TG) levels and oil red O staining showed that alcohol as well as oleate induced lipid accumulation in PHH (Figure 1A, 1B). Interestingly, the oleate and alcohol combination led to a significantly higher lipid accumulation in PHH than either of the two stimuli alone (Figure 1A, 1B).

In search for the underlying mechanisms of this synergistic effect of alcohol and oleate we analyzed the expression of fatty acid synthase (FASN) the key enzyme of hepatic *de novo* lipogenesis. Alcohol or oleate alone had slight effects on FASN mRNA levels but the alcohol/oleate combination significantly enhanced FASN expression in PHH (Figure 1C). Similarly, alcohol and oleate synergistically up-regulated the gene expression of stearoyl-CoA desaturase-1 (SCD-1), which catalyzes a rate-limiting step in the synthesis of unsaturated fatty acids, and diglyceride acyltransferase (DGAT) 1 and 2, which catalyze the final step in triglyceride formation from diacylglycerol and Acyl-CoA (Figure 1D, 1E and Suppl.Figure S2). Together, these data show a synergistic effect of oleate and alcohol stimulation on lipogenesis and cellular lipid accumulation in PHH *in vitro*.

### Effects of alcohol and oleate on hepatocellular lipid peroxidation and pro-inflammatory gene expression

**Figure 2:**
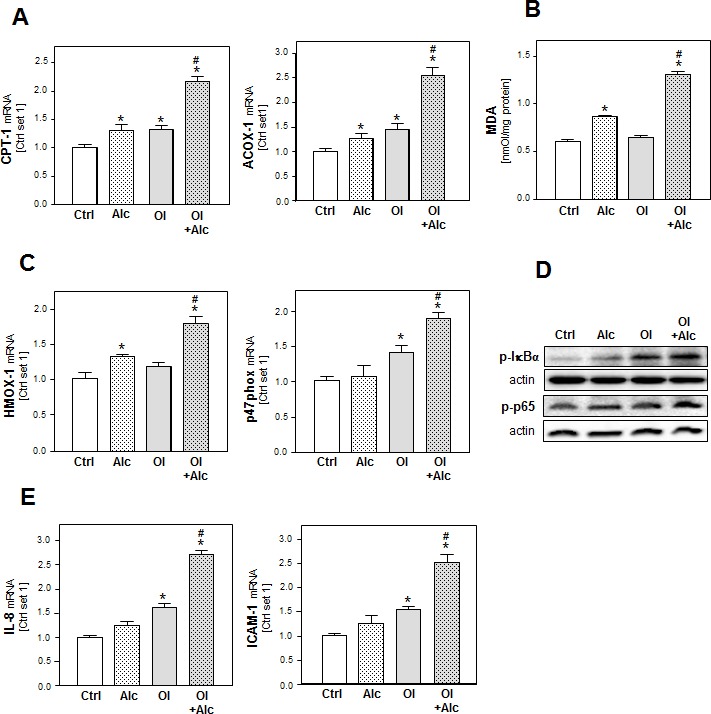
Analysis of the effect of alcohol and oleate on lipid peroxidation and pro-inflammatory gene expression PHH were pre-incubated with 0.2mM oleate (Ol) or BSA (served as control [Ctrl]) for 24h. Subsequently, cells were co-incubated with 50mM alcohol (Alc) for additional 24h. **A.** Analysis of cellular mRNA levels of CPT-1 and Acox-1 by quantitative RT- PCR. **B.** Analysis of cellular MDA levels by TBARS assay. **C.** Analysis of HMOX-1 and p47phox mRNA levels by quantitative RT-PCR. **D.** Analysis of p-IκBα and p-p65 phosphorylation by Western blot analysis. Actin served as control for loading adjustment. **E.** Analysis of IL-8 and ICAM-1 mRNA levels by quantitative RT-PCR. (*: *p* < 0.05 compared to control; #: *p* < 0.05 compared to oleate or alcohol condition).

In addition to lipid storage, enhanced lipogenesis can induce lipid combustion. Incubation with alcohol or oleate alone had only slight effects on the gene expression of carnitine palmitoyltransferase I (CPT1) and acyl-coenzyme A oxidase 1 (ACOX1), the key enzymes of the mitochondrial and peroxisomal beta-oxidation systems, respectively (Figure 2A). However, the combination significantly enhanced CPT1 and ACOX1 expression (Figure 2A) suggesting an increased oxidation rate of alcohol and free fatty acids, and consequently, an increased formation of reactive oxygen species (ROS) [24]. Accordingly, thiobarbituric acid reactive substance (TBARS) assay revealed a marked increase of malondialdehyde (MDA) levels in PHH stimulated with both alcohol and oleate while alcohol or oleate alone had no or only slight effects on cellular MDA levels (Figure 2B). Also the expression of heme oxygenase-1 (HMOX-1) and NADPH oxidase component p47phox was only significantly enhanced in PHH stimulated with the alcohol and oleate combination indicating increased cellular oxidative stress (Figure 2C). Oxidative stress is a known inducer of the transcription factor NFκB, which plays a critical role in the pathogenesis of both ALD and NAFLD [25]. NFκB is held inactive in the cytoplasm by its inhibitor IκBα, which exerts its inhibitory effect primarily through the interaction with p65, a subunit of NFκB complex, while phosphorylation of IκBα leads to its degradation, and subsequently, NFκB activation. The transcriptional activity of NFκB is controlled by phosphorylation of p65 at multiple serine residues. In our *in vitro* model, we observed enhanced phospo-IκBα and phospho-p65 levels in alcohol or oleate stimulated PHH indicative of activation of the NFκB pathway. Interestingly, phospo-IκBα and phospho-p65 levels were further enhanced by combined stimulation with both stimuli (Figure 2D). Interleukin-8 (IL-8) and intercellular adhesion molecule 1 (ICAM-1) are two pro-inflammatory genes which are regulated by NFκB and which correlate with hepatic inflammation in ALD and NAFLD [26, 27]. Notably, expression levels of IL-8 and ICAM-1 corresponded with phospo-IκBα and phospho-p65 levels in PHH stimulated with alcohol, oleate or their combination (Figure 2E). These findings indicate synergistic effects of oleate and alcohol on oxidative stress and pro-inflammatory gene expression in PHH *in vitro*.

### Role of CYP2E1 in joint effects of alcohol and oleate on hepatocellular lipid metabolism and pro-inflammatory gene expression

Ethanol metabolism by CYP2E1 leads to the generation of ROS which promote ethanol hepatotoxicity [28]. Furthermore, CYP2E1 carries out omega hydroxylation of fatty acids, and plays a critical role in the development and progression of NASH [29].

**Figure 3:**
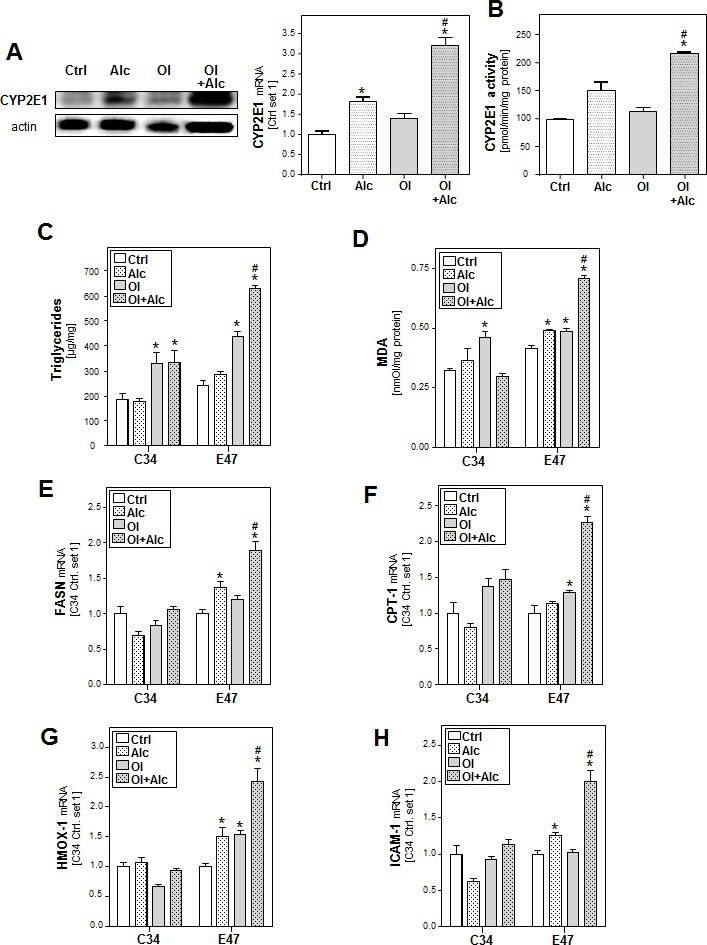
Analysis of the role of CYP2E1 in the effects of alcohol and oleate on hepatocellular lipid metabolism and pro-inflammatory gene expression PHH were pre-incubated with 0.2 mM oleate (Ol) or BSA (served as control [Ctrl]) for 24h. Subsequently, cells were co-incubated with 50mM alcohol (Alc) for additional 24h. **A.** Analysis of cellular CYP2E1 levels by quantitative RT-PCR (right panel) and Western blot analysis (left panel). **B.** Analysis of CYP2E1 activity. HepG2 E47 cells which express CYP2E1 and HepG2 C34 cells which do not express CYP2E1 were pre-incubated with 0.2 mM oleate (Ol) or BSA (served as control) for 24h. Subsequently, cells were co-incubated with 50 mM alcohol (Alc) for additional 24h. **C.** Cellular triglyceride content normalized to total cellular protein. **D.** Analysis of cellular MDA levels by TBARS assay. **E.** Analysis of cellular mRNA levels of FASN, **F.** CPT-1 **G.** HMOX1 and **H.** ICAM-1 by quantitative RT-PCR. (*: *p* < 0.05 compared to corresponding control, #: *p* < 0.05 compared to corresponding OL or alcohol condition).

In our *in vitro* system, alcohol or oleate alone only slightly increased CYP2E1 mRNA and protein expression but the combination significantly induced CYP2E1 levels compared to control cells (Figure 3A). Similar results were observed by measuring the CYP2E1 activity using p-nitrophenol as specific CYP2E1 substrate (Figure 3B).

To study the role of CYP2E1 in joint effects of alcohol and oleate, we compared hepatoma HepG2 cells expressing CYP2E1 (E47 cells) with HepG2 control cells (C34; not expressing CYP2E1) [30]. In E47 cells but not in C34 cells, combined effects of alcohol and oleate on cellular TG accumulation (Figure 3C), lipid peroxidation (Figure 3D) and gene expression of key enzymes of lipid metabolism (Figure 3E, 3F and Suppl.Figure S3A, B) and markers of oxidative stress (Figure 3G and Suppl.Figure S3C) and inflammation (Figure 3H) were significantly stronger than effects of alcohol or oleate alone.

**Figure 4:**
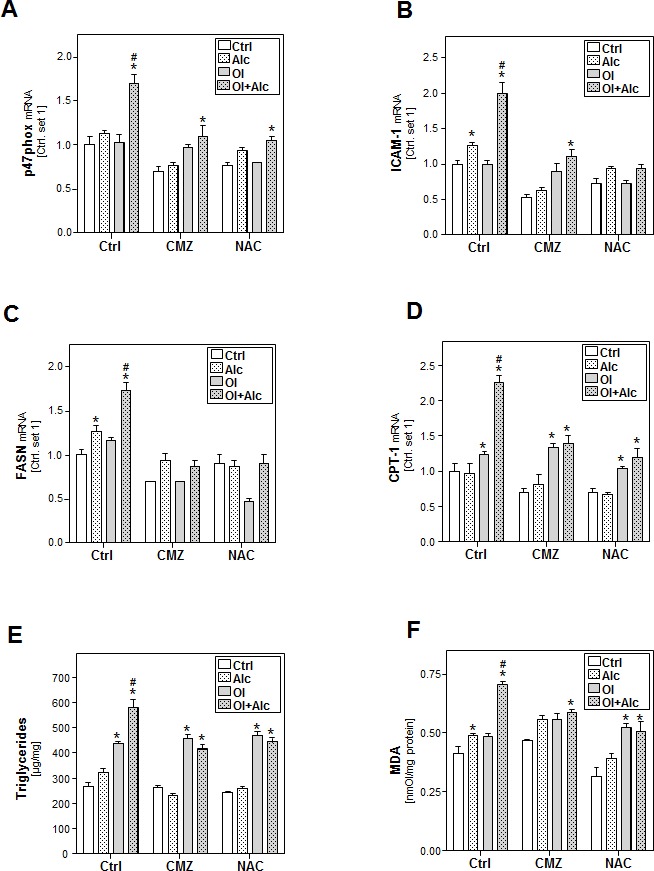
Analysis the effect of inhibition of CYP2E1 activity or ROS production on the effects of alcohol and oleate on hepatocellular lipid metabolism and pro-inflammatory gene expression HepG2 E47 cells were pre-incubated with 0.2mM oleate (Ol) or BSA (served as control) for 24 h. Subsequently, cells were co-incubated with chlormethiazole (CMZ; 100μM) or N-acetyl cysteine (NAC; 0.2mM) for 1h before adding 50mM alcohol (Alc) to cultured medium for additional 24h. **A.** Analysis of cellular mRNA levels of p47phox **B.** ICAM-1**C.** FASN and **D.** CPT-1 by quantitative RT-PCR. **E.** Cellular triglyceride content normalized to total cellular protein. **F.** Analysis of cellular MDA levels by TBARS assay. (*: *p* < 0.05 compared to corresponding control, #: *p* < 0.05 compared to corresponding oleate or alcohol condition).

To verify the central role of CYP2E1 and ROS-formation in our model, HepG2 E47 cells were incubated with chlormethiazole (CMZ), a CYP2E1 inhibitor, or N-acetyl cysteine (NAC), a ROS scavenger. Both CMZ and NAC inhibited individual as well as combined effects of alcohol and oleate on markers of oxidative stress (Figure 4A and Suppl.Figure S4A) and inflammation (Figure 4B). Also alcohol and oleate effects on FASN, SCD-1 and DGAT1/2 expression were almost completely blunted by CMZ and NAC (Figure 4C and Suppl.Figure S4B). Furthermore, CMZ and NAC inhibited both individual alcohol effects and joint alcohol/oleate effects on CPT-1 expression (Figure 4D) and cellular TG and MDA levels (Figure 4E, 4F). In contrast, individual effects of oleate on cellular lipid levels, beta-oxidation and lipid peroxidation markers were not affected by CMZ or NAC (Figure 4D-4F). According CMZ and NAC effects were observed in primary human hepatocytes (Suppl.Figure S5). Together, these data indicate that CYP2E1 and ROS-formation account for the synergistic effects of alcohol and oleate on cellular lipid accumulation, lipid peroxidation and pro-inflammatory gene expression.

### Effect of alcohol and oleate on the activation of autophagy

**Figure 5:**
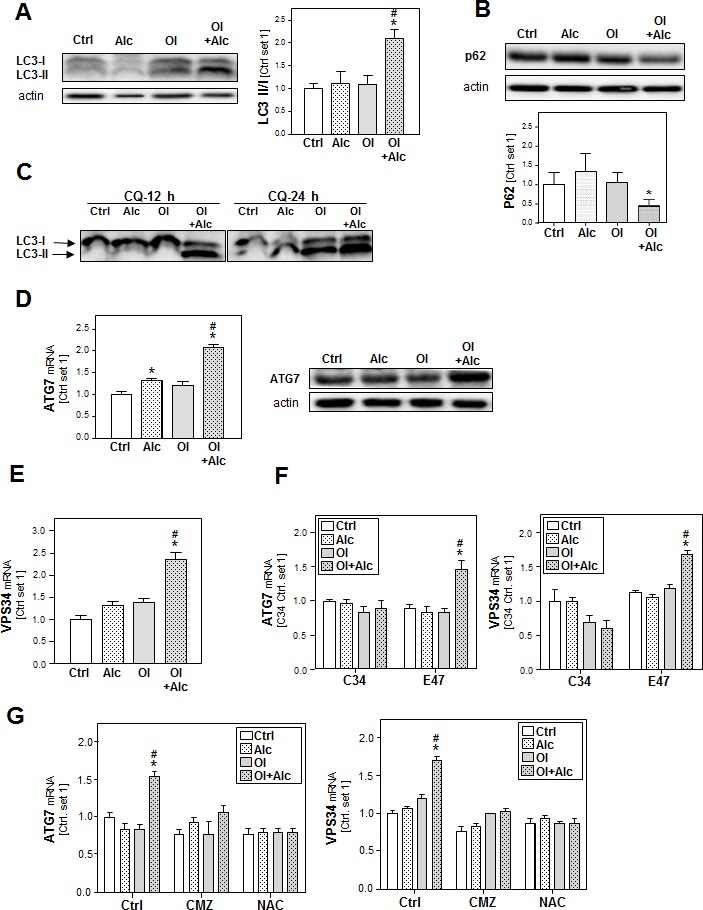
Analysis of the effect of alcohol and oleate on the activation of autophagy PHH or HepG2 E47 cells were pre-incubated with 0.2mM oleate (Ol) or BSA (served as control) for 24h. Subsequently, cells were co-incubated with/without chloroquine [CQ] (10 μM) for 1 h before adding 50 mM alcohol (Alc) to cultured medium for additional 24h. **A.** Analysis of LC3I/II protein levels in PHH by Western blot analysis (left panel); densitometric analysis of LC3II/I ratio of 3 experiments (right panel). **B.** Analysis of p62 protein expression in PHH by Western blot analysis (upper panel); densitometric analysis of p62 protein of 3 experiments (lower panel). **C.** Analysis of LC3I/II protein levels in HepG2 E47 cells by Western blot analysis. **D.** Analysis of ATG7 mRNA and protein levels in PHH by quantitative RT-PCR and Western blot analysis. Actin served as control for loading adjustment. **E.** Analysis of cellular mRNA levels of VPS34 in PHH by quantitative RT-PCR. **F.** Analysis of cellular mRNA levels of ATG7 and VPS34 by quantitative RT-PCR in HepG2 C34 (not expressing CYP2E1) in comparison with HepG2 E47 cells (expressing CYP2E1). **G.** Analysis of cellular mRNA levels of ATG7 and VPS34 by quantitative RT-PCR in HepG2 E47 cells (Ctrl cells) in comparison to chlormethiazole (CMZ) or N-acetylcysteine (NAC)-treated cells. (*: *p* < 0.05 compared to corresponding control; #: *p* < 0.05 compared to corresponding oleate or alcohol condition).

To get further insight into the joint effects of alcohol and oleate in hepatocytes, we focused on autophagy which is increasingly recognized as critical factor in the pathogenesis of both ALD and NAFLD [31, 32]. First, we investigated the effect of alcohol and oleate on the cleavage of the microtubule-associated 1 light chain 3 (LC3-I). The lipidation of LC3-I with phosphatidylethanolamine (PE) leads to the formation of LC3-II, which is a critical step in autophagosome formation and which can be visualized by western blotting where LC3-II appears as faster migrating band than LC3-I. Oleate or alcohol alone did not affect LC3-II levels but the Alc/OL combination significantly increased LC3-II expression in PHH compared to control cells (Figure 5A).

The increased LC3-II levels can be associated with either PE conjugation due to enhanced formation of autophagosomes or a block of LC3-II degradation due to impaired maturation of autophagosomes. To distinguish between these two possibilities, we detected expression levels of sequestome 1 (p62), a protein that is degraded by autophagy and accumulates when autophagy is impaired. As shown in Figure (Figure 5B), alcohol/oleate combination led to a significant decrease in p62 protein levels, which indicates increased autophagy. Pre-incubation with chloroquine (CQ) (which raises the lysosomal pH leading to inhibition of both fusion of autophagosome with lysosome and lysosomal protein degradation) caused a time-dependent increase of the LC3II/LC3I in response to stimulation with the alcohol/oleate combination in comparison to alcohol or oleate alone (Figure 5C). This indicates that the combination is causing an increased autophagic flux rather than an increase of the lysosomal activity. To assess the dynamic of the autophagic flux, we performed time course experiments and found that the alcohol/oleate combination started to induce LC3 II/I ratio after 6h. This increase became significant after 12h and continued to rise up to 24h (Suppl.Figure S6A). The raise in LC3 II/I ratio was accompanied with a concomitant reduction in p62 protein levels (data not shown).

Fitting to this, expression of the critical autophagy-related genes ATG7 and ATG12 (Figure 5D and Suppl.Figure S6B) as well as the pro-autophagic kinase vacuolar protein sorting 34 (VPS34) (Figure 5E) was not or only slightly increased in PHH after alcohol or oleate stimulation while alcohol/oleate caused a marked increase. Very similar effects were observed in CYP2E1 expressing E47 cells, while neither alcohol or oleate alone nor their combination significantly affected ATG7, ATG12 or VPS34 expression in C34 cells lacking CYP2E1 (Figure 5F and Suppl.Figure S6C). Also CMZ and NAC blunted the joint effect of alcohol and oleate on pro-autophagic gene expression (Figure 5G and Suppl.Figure S6D). Together, these findings suggest that joint effects of alcohol and oleate on CYP2E1 and ROS formation cause an induction of autophagy.

### Role of autophagy in the synergistic effects of alcohol and oleate on lipid accumulation, lipid peroxidation and hepatocellular inflammation

**Figure 6:**
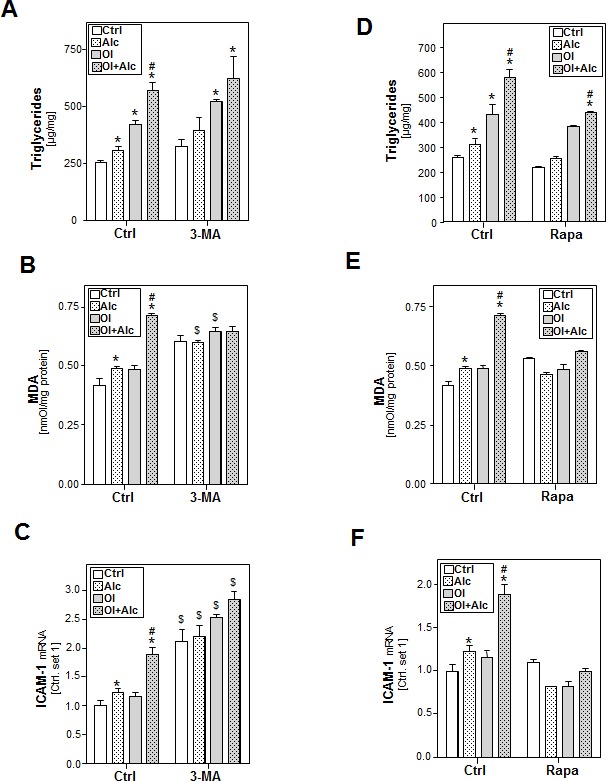
Analysis the effect of autophagy inhibition or induction on the effects of alcohol and oleate on lipid accumulation, lipid peroxidation and inflammation HepG2 E47 cells were pre-incubated with 0.2mM oleate (Ol) or BSA (served as control) for 24h. Subsequently, cells were co-incubated with 3-methyl adenine (3-MA), an autophagy inhibitor (2.5mM) or rapamycin (0.2μg/ml) for 1 h before adding 50mM alcohol (Alc) to cultured medium for additional 24 h. **A.**, **D.** Cellular triglyceride content normalized to total cellular protein. **B.**, **E.** Analysis of cellular MDA levels by TBARS assay. **C.**, **F.** Analysis of ICAM-1 mRNA levels by quantitative RT-PCR analysis. (*: *p* < 0.05 compared to corresponding control; #: *p* < 0.05 compared to corresponding oleate or alcohol condition; $: *p* < 0.05 compared to the equal condition in control group).

To assess the functional role of autophagy in synergistic alcohol and oleate mediated effects, HepG2 E47 cells were pre-incubated with 3-methyl adenine (3-MA), a highly specific inhibitor of VPS34. 3-MA treatment enhanced basal alcohol- and oleate-induced triglyceride accumulation (Figure 6A), MDA levels (Figure 6B) and ICAM-1 expression (Figure 6C). Moreover, 3-MA further enhanced the effect of the alcohol/oleate combination on the expression of the pro-inflammatory gene ICAM-1 (Figure 6C). Similarly, chloroquine treatment significantly aggravated the effects of alcohol and oleate alone as well as their combination on the expression of oxidative stress markers and pro-inflammatory genes (Supp.Figure S8). Conversely, stimulation with the autophagy inducer rapamycin slightly inhibited alcohol and oleate effects on these parameters (Figure 6D-6F). Especially, joint effects of alcohol and oleate on triglyceride, MDA and ICAM-1 mRNA levels were completely blocked by rapamycin (Figure 6D-6F). These findings could be confirmed in PHH (Suppl.Figure S7A, B). Together, these data indicate that autophagy inhibits combined alcohol and oleate mediated effects on hepatocellular lipid-accumulation, lipid-combustion and pro-inflammatory gene expression.

### The role of JNK pathway in the synergistic effects of alcohol and oleate on autophagy activation

**Figure 7:**
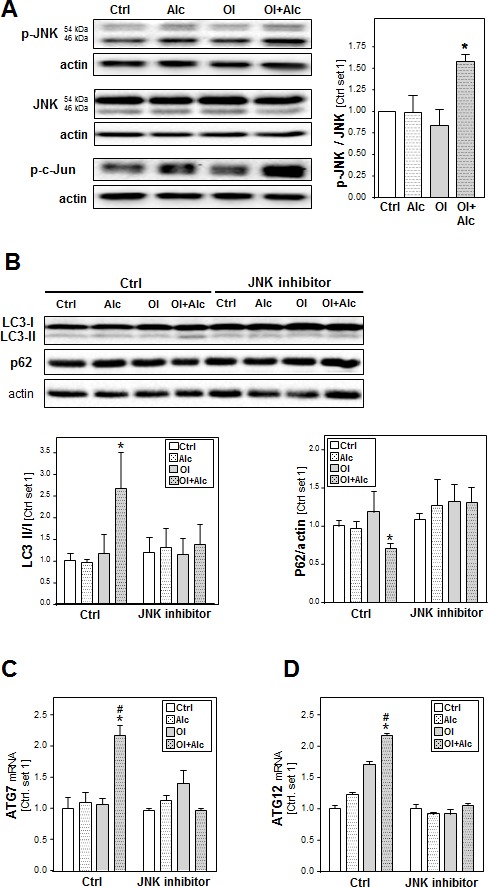
Analysis the role of JNK pathway in the synergistic effects of alcohol and oleate on autophagy activation PHH or HepG2 cells were pre-incubated with 0.2 mM oleate (Ol) or BSA (served as control) for 24 h. Subsequently, cells were co-incubated with 50 mM alcohol (Alc) for additional 24 h. **A.** Analysis of p-JNK, total JNK and p-c-JUN protein levels in PHH by Western blot analysis (left panel); densitometric analysis of p-JNK/total JNK ratio of 3 experiments (right panel). Actin served as control for loading adjustment. HepG2 E47 cells were pre-incubated with 0.2mM oleate (Ol) or BSA (served as control) for 24h. Subsequently, cells were co-incubated with JNK inhibitor (10μM) for 1 h before adding 50mM alcohol (Alc) to cultured medium for additional 24h. **B.** Analysis of LC3 I/II proteins and p62 protein levels by Western blot analysis (left panel); densitometric analysis of LC3II/LC3 I ratio (right upper panel) and p62 level (right lower panel) of 3 experiments (right panel). Actin served as control for loading adjustment. **C.** Analysis of cellular mRNA levels of ATG7 and ATG12 by quantitative RT-PCR. (*: *p* < 0.05 compared to corresponding control; #: *p* < 0.05 compared to corresponding oleate or alcohol condition).

In search for the mechanism, by which alcohol and oleate jointly affect autophagy, we analyzed the effects of these stimuli on the activation of c-Jun N-terminal kinase (JNK) because recent studies found that JNK can mediate the induction of autophagy in hepatic as well as non-hepatic cells [33-35]. Moreover, increasing oxidative stress by alcohol can activate the JNK-signaling pathway in CYP2E1-dependent manner, and also free fatty acids have been shown to induce autophagy in hepatocytes *via* JNK activation [34, 36, 37]. In our experimental model, stimulation with alcohol or oleate alone exhibited no or only slight effects on phopsho-JNK and phospho-c-JUN protein levels in PHH while the combination caused a significant increase of JNK and c-JUN phosphorylation (Figure 7A). Neither alone nor in combination alcohol and oleate affected the total JNK expression levels (Figure 7A). Pre-incubation with the specific JNK inhibitor (SB600125) abrogated the alcohol- and oleate-induced increase of LC3-II levels as well as the reduction in p62 protein levels in hepatocytes (Figure 7B). Moreover, JNK inhibition blocked the alcohol/oleate-induced ATG7 and ATG12 up-regulation (Figure 7C, 7D). Together, these findings indicate that the joint effects of alcohol and oleate on autophagy in hepatocytes are induced *via* JNK-activation.

## DISCUSSION

In this study, we developed a new *in vitro* model to study the effects of moderate, non-toxic alcohol levels on lipid-loaded and control hepatocytes and unraveled interacting, synergistic effects between hepatocellular lipid accumulation and alcohol metabolism.

Circulating free fatty acids (FFA) are also the major mediators of excessive hepatic lipid accumulation in patients with NAFLD. The rate of hepatic FFA uptake is not regulated, and therefore, is proportional to plasma FFA concentrations [38]. In NAFLD patients, circulating FFA are commonly elevated, and their plasma levels correlate with disease severity [39]. Already previously, we had established an *in vitro* model of cellular lipid accumulation in PHH and HepG2 cells and confirmed that the pathological mechanisms observed in this model system closely mimic the pathological alterations found in hepatic tissue of NAFLD-patients [19, 20, 40].

In the present study, we combined this model with alcohol stimulation. Also alcohol is known to induce hepatocellular lipid accumulation *in vitro* and *in vivo* [23, 41]. Alcohol oxidation leads to acetyl-CoA synthesis from acetate or citrate that constitutes the substrates for lipogenic enzymes, and accordingly, we observed an increased expression of FASN and SCD1 in alcohol stimulated PHH. Also stimulation with oleate caused an increase in the expression of both enzymes but this induction was significantly stronger in combination with alcohol. Similarly, the induction of triglyceride synthesis and cellular triglyceride levels were moderate upon stimulation with alcohol or oleate compared to the combination of both stimuli. Similar joint effects were observed on the expression of key enzymes of lipid oxidation, which are known to cause ROS formation. Accordingly, also gene expression indicative for oxidative stress and cellular malondialdehyde (MDA) levels were markedly increased by alcohol in combination with oleate stimulation while either of the two stimuli alone exhibited no or only slight effects. Moreover, we found that alcohol and oleate combination induced significantly more pro-inflammatory activity in hepatocytes than either of the two stimuli alone. Previous studies have found that effects of free fatty acids on hepatocytes may dependent on their degree of saturation [42, 43]. In the present study we mainly applied the unsaturated fatty acid oleate to induce hepatocellular lipid accumulation because we have previously shown that the oleate induced *in vitro* steatosis causes cellular alterations similar to those found in hepatic tissue of NAFLD-patients [19, 20]. However, we also analyzed the combined effects of alcohol and the saturated fatty acid palmitate on the expression of genes that affect Δ-oxidation, autophagy and inflammation and found similar effects as observed by alcohol in combination with oleate (Suppl.Figure S9).

Noteworthy, joint effects of alcohol and oleate on cellular lipid accumulation and pro-inflammatory gene expression were dependent on CYP2E1 activity. CYP2E1 activity has been shown to correlate with alcohol-induced liver injury, and inhibition of CYP2E1 prevented the induction of hepatic steatosis and ROS production in models of alcoholic steatohepatitis [23, 41, 44]. Also in NASH, increased CYP2E1 activity has been described and has been identified as a pathogenic factor [45-47]. Interestingly, in our *in vitro* model we found that the combination of alcohol and oleate induced CYP2E1 levels and its activity significantly more than each stimulus alone. Moreover, CYP2E1 inhibition had only slight effects on hepatocellular lipid accumulation and MDA formation induced by singly alcohol or oleate stimulation, respectively. In contrast, joint effects of alcohol and oleate on hepatic steatosis and lipid peroxidation were completely blunted. These effects indicate a crucial role of CYP2E1 and subsequent ROS production in the underlying pathophysiological mechanism of synergistic effects of alcohol and oleate.

Autophagy, a highly conserved intracellular catabolic pathway for the degradation of long-lived proteins and cytoplasmic organelles, can either be increased or decreased by ethanol depending on the used model, the dose, the evaluated tissue and the experimental conditions [48]. While the effects of ethanol on autophagy are complex and require further study, it is becoming clear that autophagy serves a protective function against ethanol-induced liver injury [31]. Moreover, induction of autophagy has been shown to alleviated hepatic steatosis and injury in models of acute and chronic ethanol exposure [23, 31, 36, 49]. Of note, ethanol-induced autophagy requires its metabolism *via* CYP2E1 and subsequent ROS production [23, 36, 50]. Moreover, blocking CYP2E1 or inhibiting ROS by antioxidants also diminished GFP-LC3 puncta [23, 36, 50]. It seems that alcohol oxidation by CYP2E1 is also important for alcohol-induced inhibition of cellular proteasome activity and increased autophagosome numbers [51].

In our *in vitro* model, which mimics moderate alcohol exposure and FFA-induced steatosis, neither alcohol nor oleate alone caused a significant induction of autophagy markers in hepatocytes. In contrast, the alcohol/oleate combination led to a marked up-regulation of the autophagic flux, and we revealed that this synergistic effect on autophagy was mediated *via* JNK-activation. Inhibition of autophagy induced lipid accumulation, oxidative stress and inflammation, while induction of autophagy alleviated these mechanisms. Still, it appears that under the experimental conditions used, autophagy could not counterbalance the negative effects in this *in vitro* model. One may speculate that also *in vivo* there is a fine balance between protective and damaging mechanisms. Only slight differences in the ratio of individual beneficial and detrimental factors may decide whether moderate alcohol consumption is protective or is causing harm in (non-alcoholic) fatty livers. Actually, there are some studies assuming that moderate alcohol consumption might even be protective for patients with NAFLD [52, 53]. It is intriguing to speculate whether intensified effects of alcohol on autophagy in steatotic hepatocytes may (in part) account for such observations.

Of note, the joint effect of alcohol and cellular steatosis on autophagy required CYP2E1 activity in our *in vitro* model. Generally, this cytochrome P450 is known for its detrimental effects in alcoholic liver disease through free radical formation and lipid peroxidation [23, 54]. Therefore, pharmacological inhibition of CYP2E1 has emerged as strategy for treatment of alcohol-induced liver injury [54, 55]. Also in our *in vitro* system, CYP2E1 accounts for detrimental effects of alcohol in lipid loaded hepatocytes, and certainly, the clinical relevancy of our *in vitro* findings has to be verified. Still, the CYP2E1 mediated joint effect of alcohol- and oleate-induced steatosis on autophagy indicate that the manipulation of CYP2E1 may be a double-edged sword and warrant the exercise of caution in the pharmacological use of CYP2E1 inhibitors for the treatment of alcoholic liver disease in obese individuals. Furthermore, one may speculate whether individual factors tipping the balance on the one or the other side of detrimental or beneficial joint effects of alcohol and free fatty acids account at least in part for the high variation in the clinical course of alcoholic liver disease.

In summary, in our *in vitro* model, exposure of hepatocytes to alcohol and the fatty acid oleate caused a synergistic effect on lipid accumulation, lipid peroxidation, oxidative stress and inflammatory gene expression. Conversely, alcohol and oleate jointly induced autophagy which reduced these pathological mechanisms. Noteworthy, both detrimental as well as beneficial joint effect of alcohol and FFA were dependent on CYP2E1 activity. Upon clinical verification these *in vitro* findings may have important implications on the prognosis or treatment of alcoholic liver disease.

## MATERIALS AND METHODS

### Cells and cell culture model

Isolation and culture of primary human hepatocytes (PHH) were performed as described [56]. Human liver tissue for cell isolation was obtained from the charitable state controlled foundation HTCR, with informed patient consent and approved by the local Ethics Committee.

HepG2 E47 cells which express CYP2E1 and HepG2 C34 cells which do not express CYP2E1 were cultured as described [30].

PHH or HepG2 cells were incubated with oleate (Ol) (doses up to 0.2 mM) for 24 h as described [20]. We have previously shown that this treatment leads to cellular steatosis and pathological alterations similar to those found in hepatic tissue of NAFLD-patients [19, 20, 40]. Subsequently, cells were co-incubated with clinically relevant non-toxic concentration of alcohol (Alc) (up to 50 mM) [21-23] for 16-24 h. In additional experimental conditions, cells were treated with 3-methyl adenine (3-MA; 2.5 mM), rapamycin (0.2 μg/ml), chlormethiazole (CMZ; 100 μM), N-acetyl cysteine (NAC; 0.2 mM), JNK inhibitor (SB600125; 10 μM) or chloroquine (CQ; 10 μM) for 1 h before adding alcohol to the cell cultured medium. All experiments have been repeated at least three times.

### Mitochondrial activity assay

For quantification of hepatocellular mitochondrial activity, the colorimetric XTT assay (Roche Diagnostics, Mannheim, Germany) was used according to the manufacturer's instructions.

### Analysis of CYP2E1 activity

Hepatocytes were washed twice, incubated with 0.2 mM *p*-nitrophenol at 37°C for 2h, and then the reaction was terminated by adding trichloroacetic acid to a final concentration of 1% (v/v). Cells were harvested and centrifuged at 5,000*g* for 5 min, and the supernatants were assayed for 4-nitrocatechol by adding NaOH to a final concentration of (1N) and immediately determining the absorbance at 492 nm.

### Analysis of cellular lipid content

Cellular lipid droplets were visualized by Oil Red O staining as described [20]. Lipid droplets were assessed using a light microscope with digitalized camera and a Metaview image analysis system (Olympus America Inc., PA). The density of lipid droplets per microscopic area was calculated from four different fields. Total cellular triglycerides were extracted and quantified by the triglyceride determination kit (GPO) (Sigma, Deisenhofen, Germany) as described [20].

### Analysis of lipid peroxidation

Cellular malondialdehyde (MDA) levels were analyzed using the OxiSelect-thiobarbituric acid-reactive substance (TBARS) assay kit (Cell Biolabs, San Diego, CA, USA) following the manufacturer's instructions.

### Quantitative real-time-PCR analysis

RNA isolation from cultured cells and reverse transcription were performed as described [57]. Quantitative real-time-PCR was performed applying LightCycler technology (Roche) [57] with specific sets of primers. Amplification of cDNA derived from 18s rRNA was used for normalization.

### Protein analysis

Protein extraction and Western blotting was performed as described [58] applying anti-rabbit antibodies against phospho-IκBα (#2859), phospho-NFκBα p65 (#3033), phospho-JNK (#9251), JNK (#9258), phospho-c-Jun (#3270), p62 (#5114) and ATG7 (#2631), all from Cell Signaling Technology (Danvers, MA, USA; all diluted 1:1,000). Furthermore, antibody against LC3 (NB100-2220, Novus Biologicals, Cambridge, UK; 1:1000) was applied.

### Statistical analysis

Values are presented as mean ± SEM. Comparison between groups was made using the Student's unpaired t-test. A p value <0.05 was considered statistically significant. All calculations were performed using the statistical computer package GraphPad Prism version 4.00 for Windows (GraphPad Software, San Diego, USA).

## SUPPLEMENTARY MATERIAL FIGURES


