# Supplementary material for: Identification of cytochrome CYP2E1 as critical mediator of synergistic effects of alcohol and cellular lipid accumulation in hepatocytes in vitro
Source: Oncotarget. 2015 Oct 20;6(39):41464–78. doi: 10.18632/oncotarget.6203 (PMC4747167; doi:10.18632/oncotarget.6203)
Supplement: Supplementary file 1 [file oncotarget-06-41464-s001.pdf]

Identification of cytochrome CYP2E1 as critical mediator of synergistic effects of alcohol and cellular lipid accumulation in hepatocytes *in vitro*

Supplementary Material

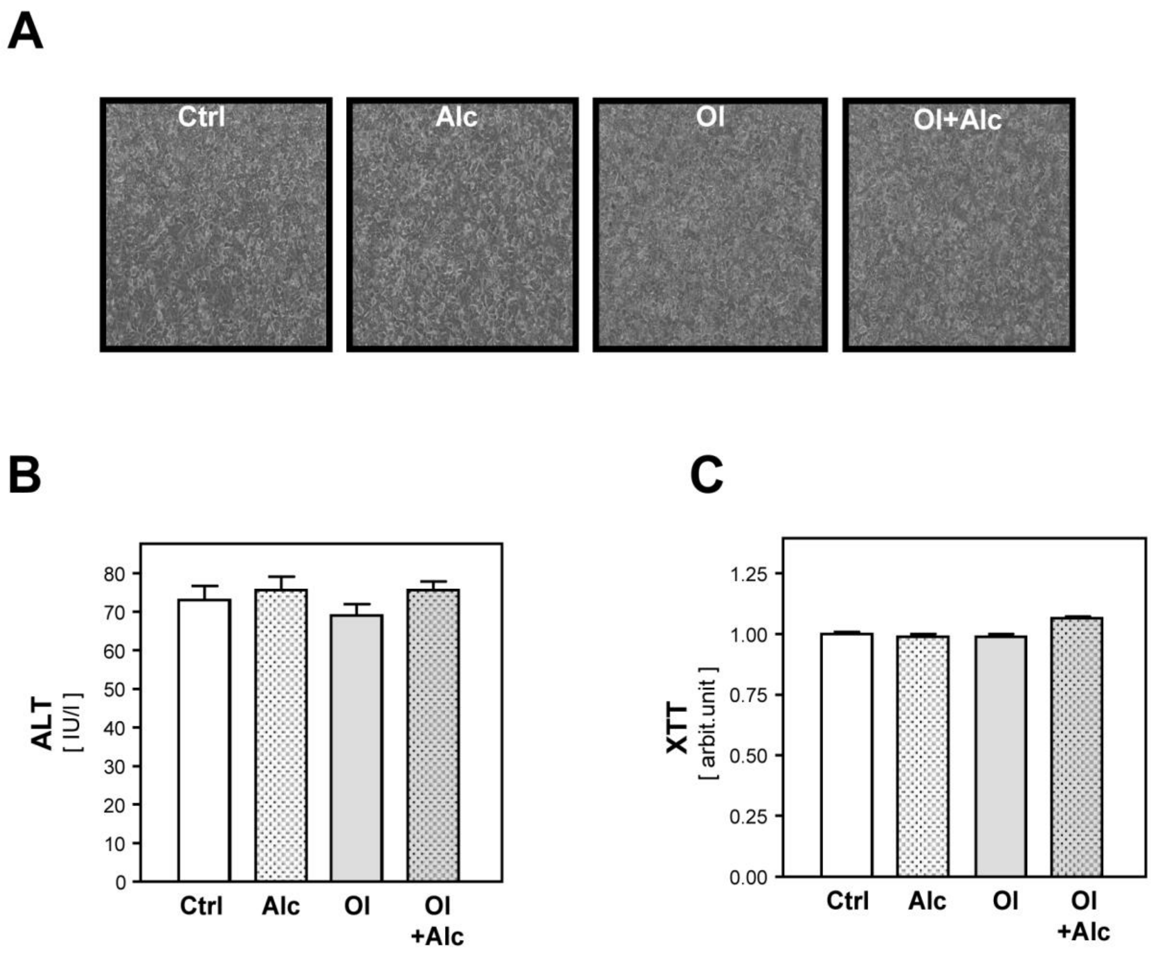

Figure S1: Analysis the cytotoxic effects of alcohol and oleate on primary human hepatocytes (PHH)

PHH were pre-incubated with 0.2 mM oleate (Ol) or BSA (served as control) for 24 h. Subsequently, cells were co-incubated with 50 mM alcohol (Alc) for additional 24 h.

(A) Microscopic images.

(B) ALT leakage.

(C) XTT activity

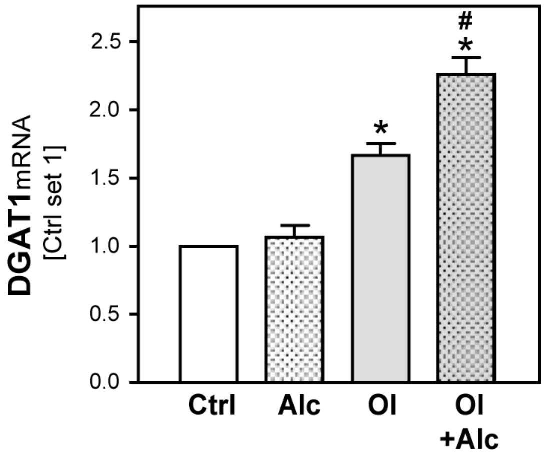

Figure S2: Analysis of the effect of alcohol and oleate on hepatocellular lipid metabolism and lipid accumulation

PHH were pre-incubated with 0.2 mM oleate (Ol) or BSA (served as control) for 24 h. Subsequently, cells were co-incubated with 50 mM alcohol (Alc) for additional 24 h for mRNA analysis of DGAT1.

(*: $p < 0.05$ compared to control, #: $p < 0.05$ compared to oleate or alcohol condition).

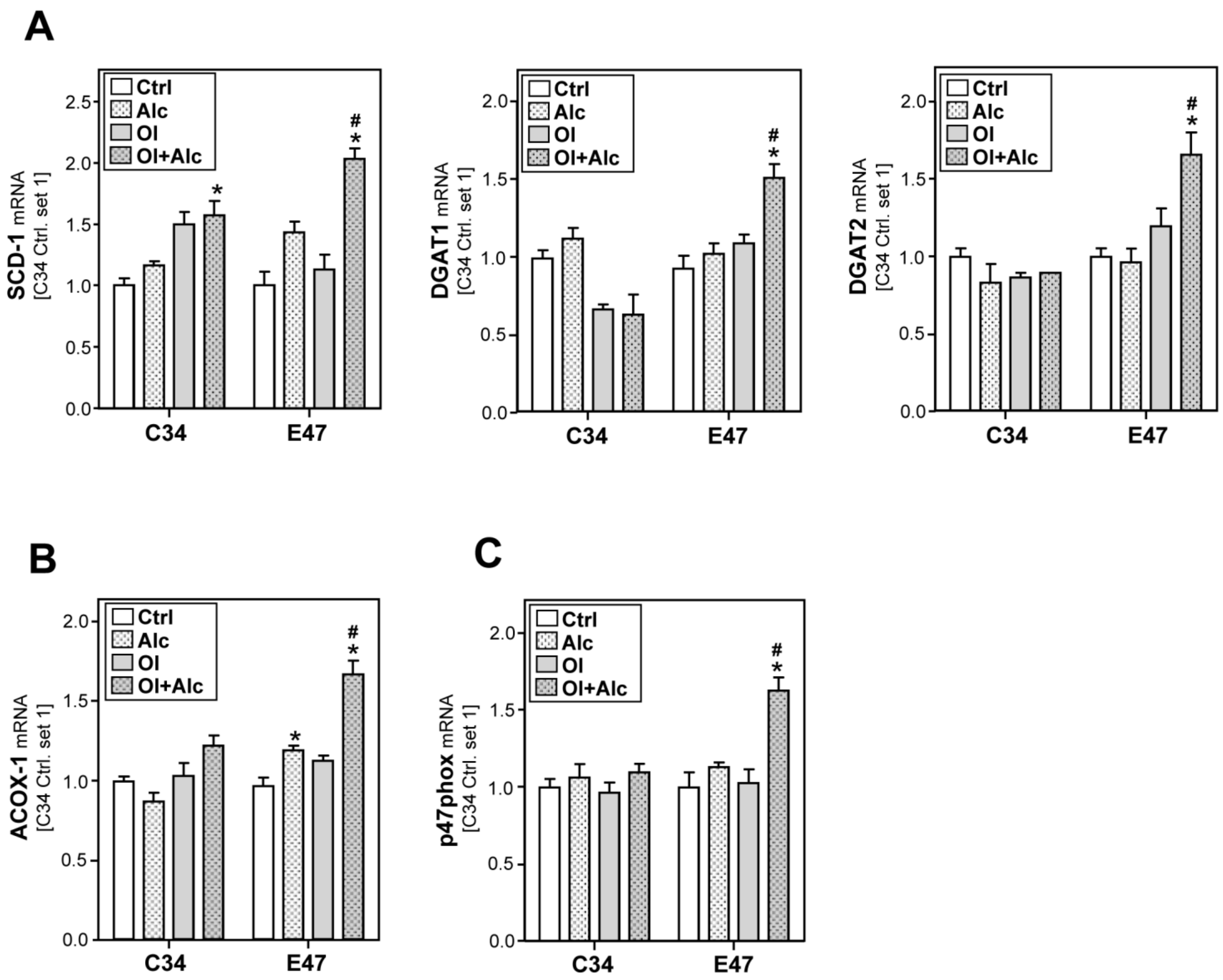

Figure S3: Analysis of the effect of alcohol and oleate on lipid peroxidation and pro-inflammatory gene expression

HepG2 E47 cells which express CYP2E1 and HepG2 C34 cells which do not express CYP2E1 were pre-incubated with 0.2 mM oleate (Ol) or BSA (served as controls [Ctrl]) for 24h. Subsequently, cells were co-incubated with 50mM alcohol (Alc) for additional 24h for mRNA analysis of **(A)** SCD-1, DGAT1 and DGAT2 **(B)** ACOX-1 **(C)** p47phox by quantitative RT-PCR.

(*: p< 0.05 compared with corresponding control, #: p< 0.05 compared with corresponding oleate or alcohol).

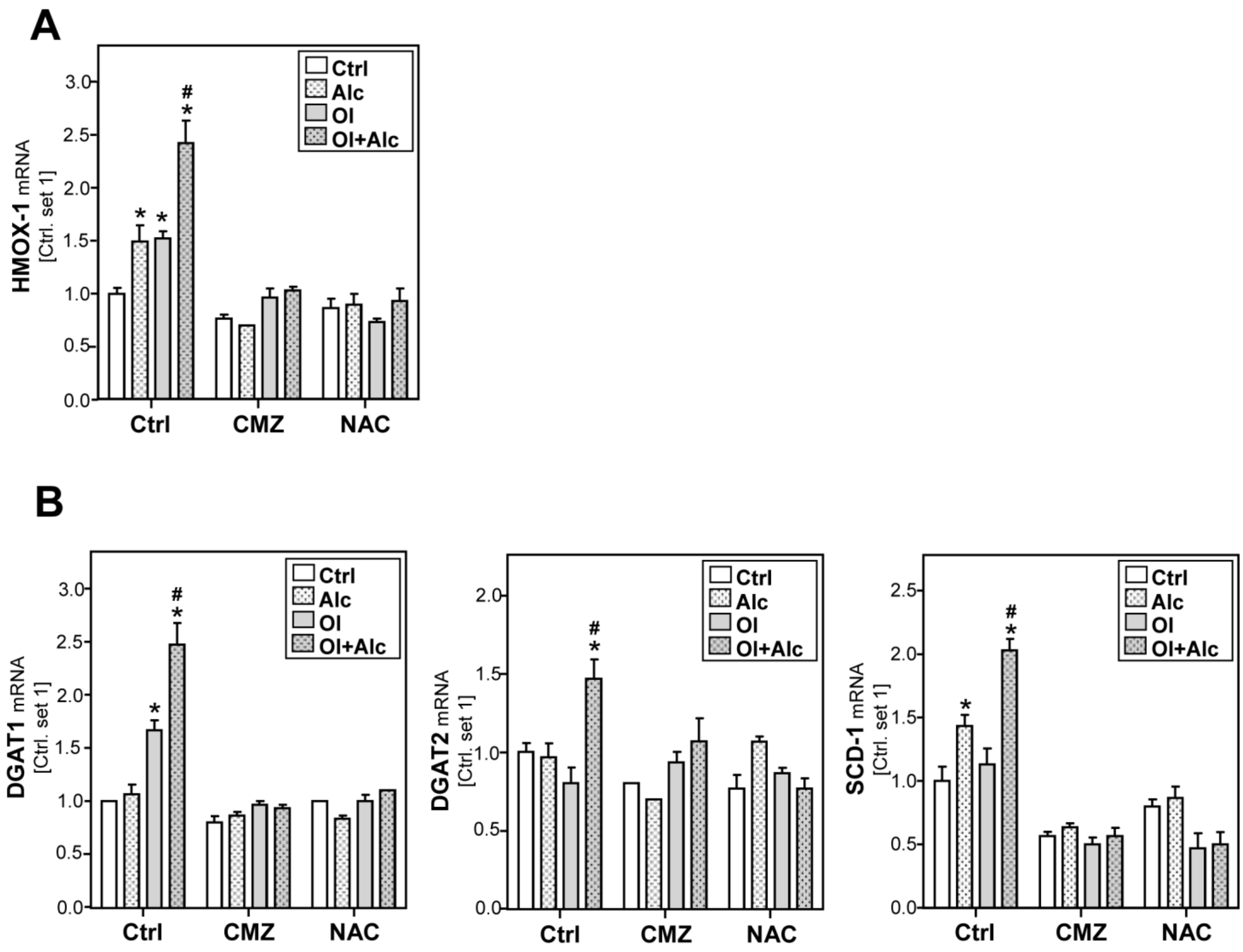

Figure S4: Analysis the effect of inhibition of CYP2E1 activity or ROS production on the effects of alcohol and oleate on hepatocellular lipid metabolism and pro-inflammatory gene expression

HepG2 E47 cells which express CYP2E1 were pre-incubated with 0.2 mM oleate (Ol) or BSA (served as control) for 24 h. Subsequently, cells were co-incubated with Chlormethiazole (CMZ), a CYP2E1 inhibitor (100 µM) or N-acetyl cystein (NAC), a ROS scavenger (0.2 mM) for 1 h before adding 50 mM alcohol (Alc) to cultured medium for additional 24 h. Analysis of mRNA levels of (**A)** HMOX-1 **(B)** DGAT1, DGAT2 and SCD-1 by quantitative RT-PCR.

(*: $p < 0.05$ compared to corresponding control, #: $p < 0.05$ compared to corresponding Ol or alcohol condition).

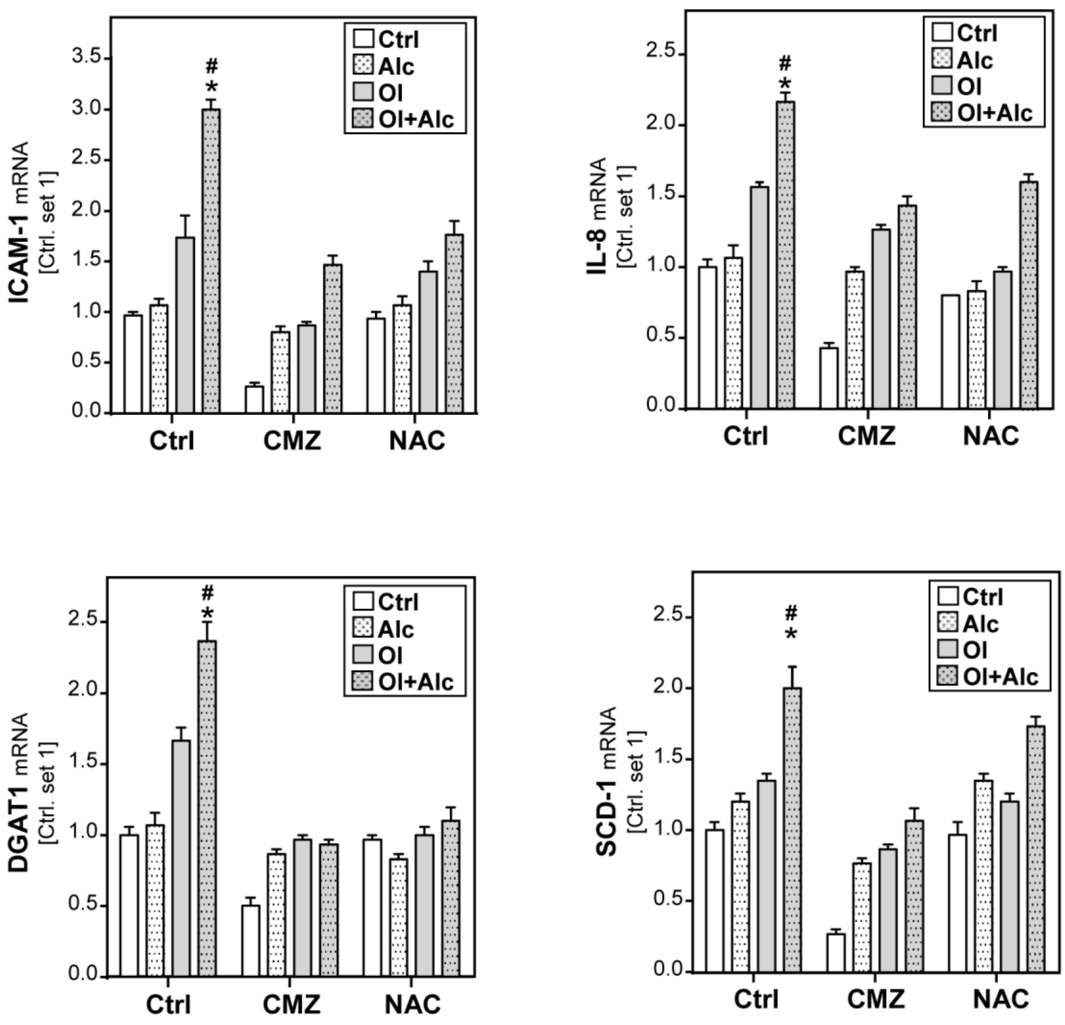

Figure S5: Analysis the effect of inhibition of CYP2E1 activity or ROS production on the effects of alcohol and oleate on hepatocellular lipid metabolism and pro-inflammatory gene expression

PHH cells were pre-incubated with 0.2 mM oleate (Ol) or BSA (served as control) for 24 h. Subsequently, cells were co-incubated with Chlormethiazole (CMZ), a CYP2E1 inhibitor (100 µM) or N-acetyl cystein (NAC), a ROS scavenger (0.2 mM) for 1 h before adding 50 mM alcohol (Alc) to cultured medium for additional 24 h. Analysis of mRNA levels of ICAM-1, IL-8, DGAT1 and SCD-1 by quantitative RT-PCR.

(*: $p < 0.05$ compared to corresponding control, #: $p < 0.05$ compared to corresponding oleate or alcohol condition).

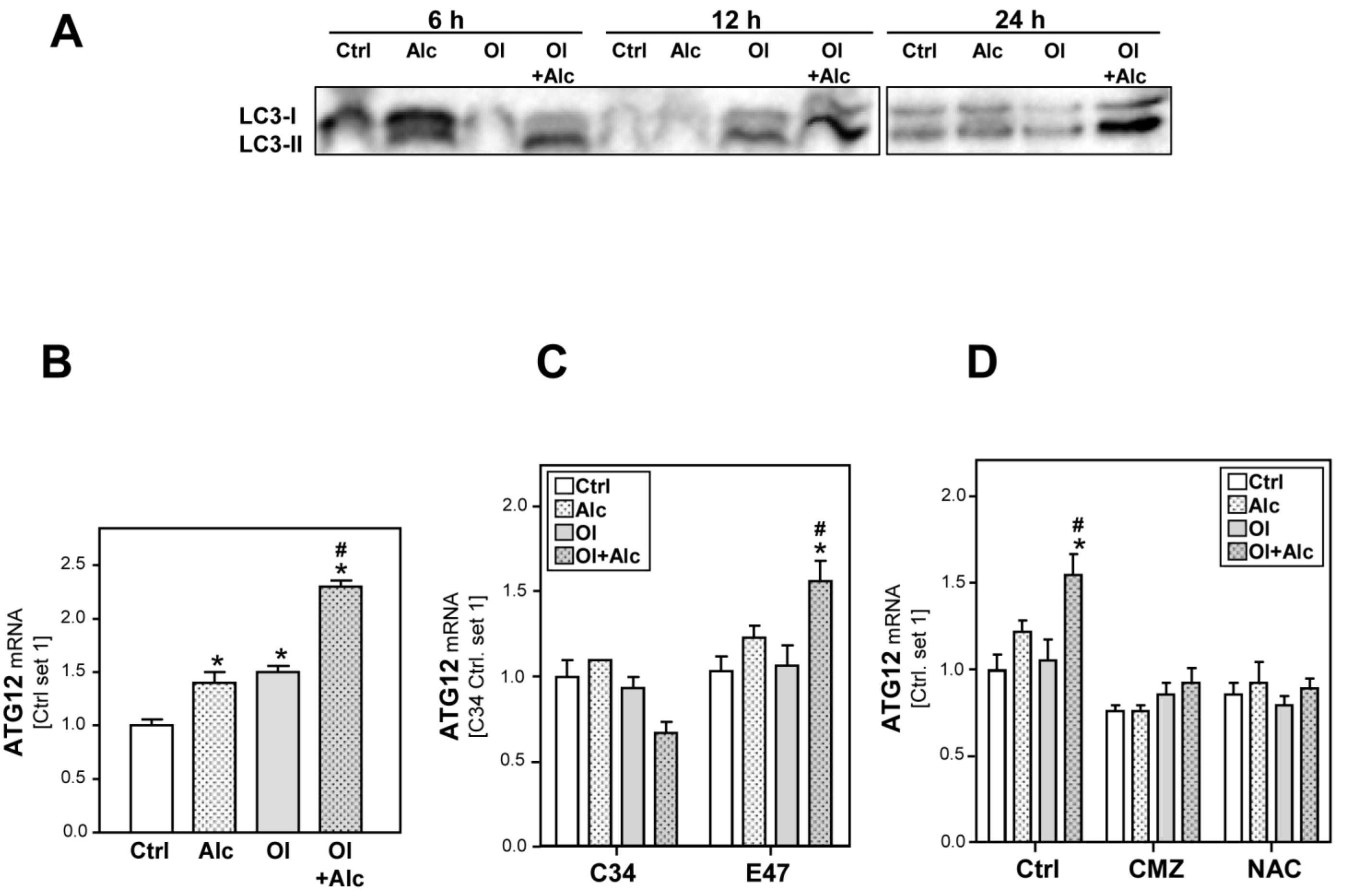

Figure S6: Analysis of the effect of alcohol and oleate on hepatocellular autophagy

HepG2 E47 cells were pre-incubated with 0.2 mM oleate (Ol) or BSA (served as control) for 24 h. Subsequently, cells were co-incubated with 50mM alcohol (Alc) for additional 6,12 and 24 h.

(A) Analysis of LC3 I/II protein levels by Western blot analysis..

(B) PHH were pre-incubated with 0.2 mM oleate (Ol) or BSA (served as control) for 24 h. Subsequently, cells were co-incubated with 50 mM alcohol (Alc) for additional 24 h for mRNA analysis of ATG12.

(C) HepG2 E47 cells which express CYP2E1 and HepG2 C34 cells which do not express CYP2E1 were were pre-incubated with 0.2 mM oleate (Ol) or BSA (served as controls) for 24 h. Subsequently, cells were co-incubated with 50 mM alcohol (Alc) for additional 24 h for mRNA analysis of ATG12.

(D) HepG2 E47 cells which express CYP2E1 were pre-incubated with 0.2 mM oleate (Ol) or BSA (served as control) for 24 h. Subsequently, some cells were co-incubated with Chlormethiazole (CMZ), a CYP2E1 inhibitor (100 µM) or N-acetyl cystein (NAC), a ROS scavenger (0.2mM) for 1 h before adding 50 mM alcohol (Alc) to cultured medium for additional 24 h for mRNA analysis of ATG12.

(*: $p < 0.05$ compared to corresponding control, #: $p < 0.05$ compared to corresponding oleate or alcohol condition).

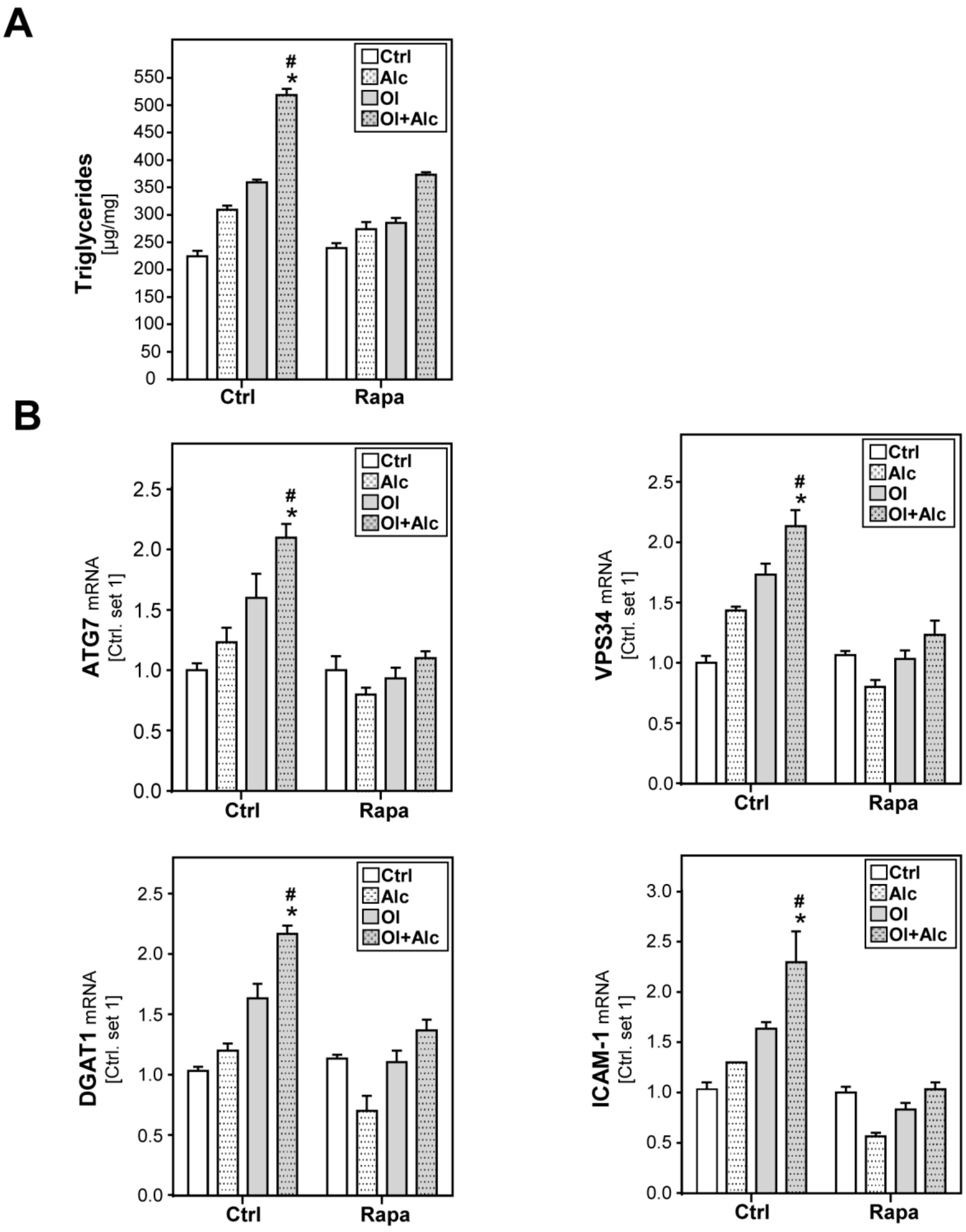

Figure S7: Analysis the effect of autophagy induction on the effects of alcohol and oleate on lipid metabolism and pro-inflammatory and pro-autophagic gene expression

PHH cells were pre-incubated with 0.2 mM oleate (Ol) or BSA (served as control) for 24 h. Subsequently, cells were co-incubated with rapamycin, an autophagy inducer (0.2 µg/ml) for 1 h before adding 50 mM alcohol (Alc) to cultured medium for additional 24 h.

(A) Cellular triglyceride content normalized to total cellular protein.

(B) Analysis of ATG7, VPS34, DGAT1 and ICAM-1 mRNA levels by quantitative RT-PCR analysis.

(*: $p< 0.05$ compared to corresponding control; #: $p< 0.05$ compared to corresponding oleate or alcohol condition).

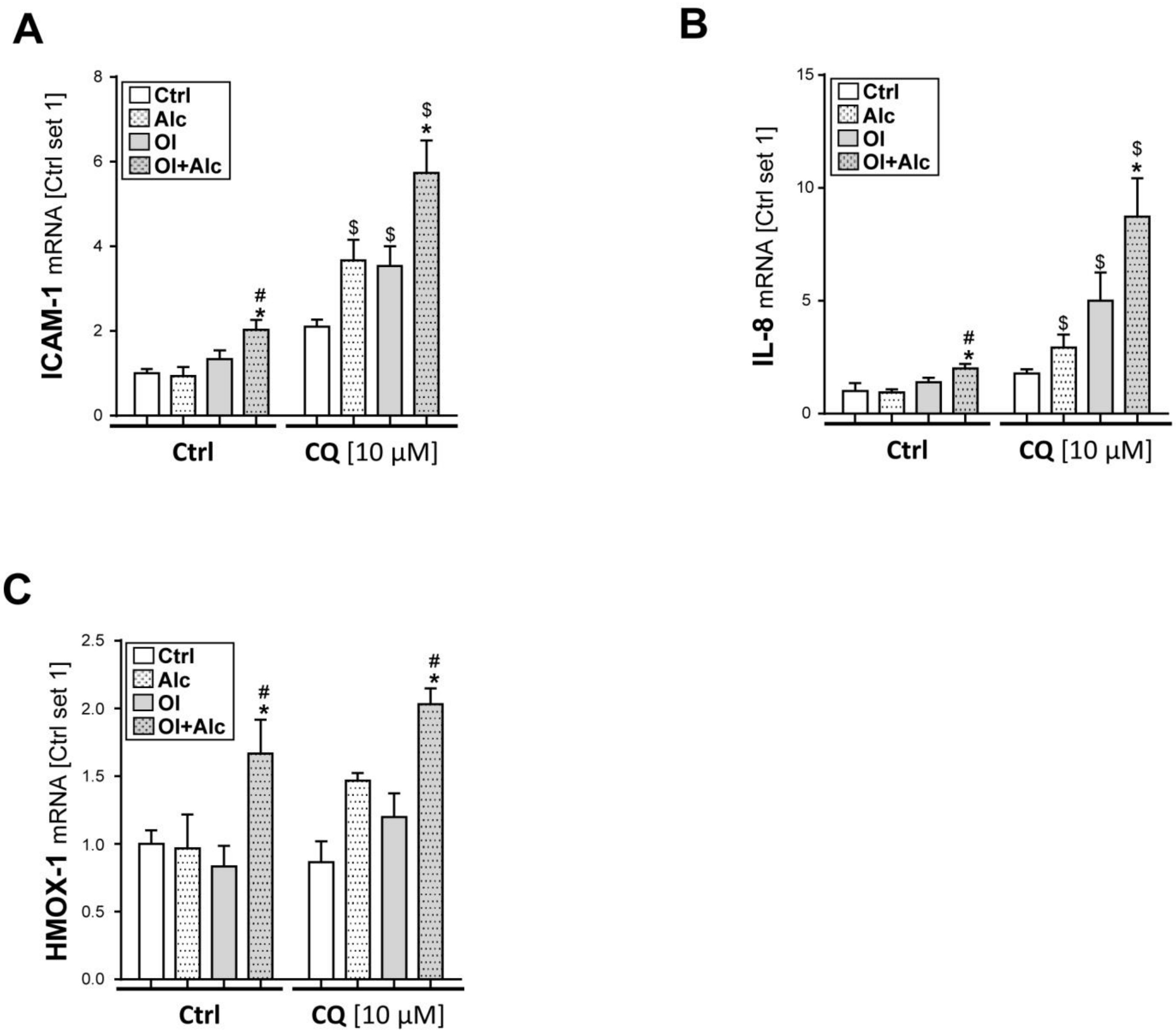

Figure S8: Analysis the effect of autophagy inhibition on the effects of alcohol and oleate on oxidative stress and inflammation markers

HepG2 E47 cells were pre-incubated with 0.2 mM oleate (Ol) or BSA (served as control) for 24 h. Subsequently, cells were co-incubated with/without chloroquine [CQ] (10 µM) for 1 h before adding 50 mM alcohol (Alc) to cultured medium for additional 24h for mRNA analysis of **(A)** ICAM-1**(B)** IL-8 **(C)** HMOX-1 and by quantitative RT-PCR.

(*: $p < 0.05$ compared with corresponding control, #: $p < 0.05$ compared with corresponding oleate or alcohol, $: $p < 0.05$ compared to the equal condition in control group).

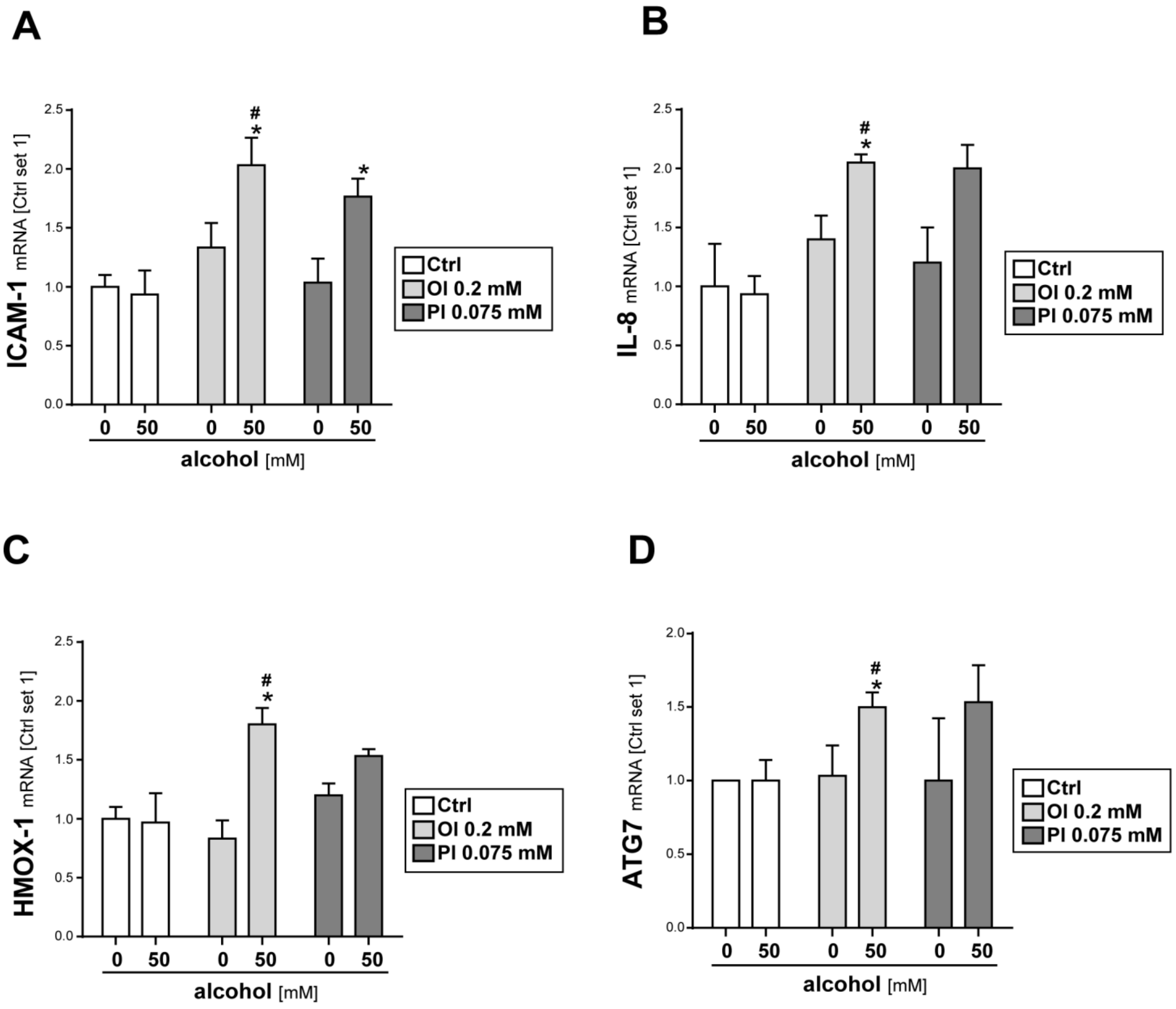

Figure S9: Analysis of the effect of alcohol and oleate or palmitate on oxidative stress, pro-inflammatory and pro-autophagic gene expression

HepG2 E47 cells which express CYP2E1 were pre-incubated with 0.2 mM oleate (Ol) or 0.075 mM palmitate (Pl) or BSA (served as controls [Ctrl]) for 24h. Subsequently, cells were co-incubated with 50mM alcohol (Alc) for additional 24h for mRNA analysis of **(A)** ICAM-1**(B)** IL-8 **(C)** HMOX-1 and **(D)** ATG7 by quantitative RT-PCR.

(*: $p< 0.05$ compared with corresponding control, #: $p< 0.05$ compared with corresponding oleate or alcohol).
